# Award to Messrs. Billings, Clapp & Co., Boston

**Published:** 1877-04

**Authors:** 


Award to Messrs. Billings, Clapp & Co., Boston.—The undersigned, having examined the products herein described, respectfully recommend the same to the United States Centennial Commission for Award, for the following reasons, namely:

A very fine display of chemicals, *especially* carbolic acid, propylamine [trimethylamine], chloride of propylamine, and also of pharmaceutical chemicals, such as citrates of iron and quinia, citrates of iron and magnese, citrates of bismuth and ammonium, pyrophosphate of iron, bromide of potassium, bromide of ammonium, chromic acid, valerianic acid, and many others. Commended for display and *excellence* of chemicals.

F. A. Genth.

[Signature of the Judge.]

*Approval of Group of Judges.*

| | |
|---|---|
| J. Lawrence Smith, | Dr. V. Wagner. |
| P. De Wilde, | Charles A. Joy, |
| E. Paterno, | J. W. Mallet, |
| F. Kuhlman. | |

